# Supplementary material for: Scoring epidemiological forecasts on transformed scales
Source: PLoS Comput Biol. 2023 Aug 29;19(8):e1011393. doi: 10.1371/journal.pcbi.1011393 (PMC10495027; doi:10.1371/journal.pcbi.1011393)
Supplement: S1 Text — (PDF) [file pcbi.1011393.s001.pdf]

Instead of defining the WIS as an average of scores for individual quantiles, we can define it using an average of scores for symmetric predictive intervals. For a single prediction interval, the interval score (IS) is computed as the sum of three penalty components, dispersion (width of the prediction interval), underprediction and overprediction,

$$
\begin{aligned}
IS_\alpha(F, y) &= (u - l) + \frac{2}{\alpha} \cdot (l - y) \cdot \mathbf{1}(y \leq l) + \frac{2}{\alpha} \cdot (y - u) \cdot \mathbf{1}(y \geq u) && (1) \\
&= \text{dispersion} + \text{underprediction} + \text{overprediction}, && (2)
\end{aligned}
$$

where $\mathbf{1}()$ is the indicator function, y is the observed value, and l and u are the $\frac{\alpha}{2}$ and $1 - \frac{\alpha}{2}$ quantiles of the predictive distribution, i.e. the lower and upper bound of a single central prediction interval. For a set of K^* prediction intervals and the median m, the WIS is computed as a weighted sum,

$$
\text{WIS} = \frac{1}{K^* + 0.5} \cdot \left(w_0 \cdot |y - m| + \sum_{k=1}^{K^*} w_k \cdot IS_{\alpha_k}(F, y) \right), \quad (3)
$$

where w_k is a weight for every interval. Usually, $w_k = \frac{\alpha_k}{2}$ and $w_0 = 0.5$.
