# Supplementary material for: Scoring epidemiological forecasts on transformed scales
Source: PLoS Comput Biol. 2023 Aug 29;19(8):e1011393. doi: 10.1371/journal.pcbi.1011393 (PMC10495027; doi:10.1371/journal.pcbi.1011393)
Supplement: S1 Table — (PDF) [file pcbi.1011393.s002.pdf]

target type	quantity	measure	natural	log
Cases	Observations	mean	61979	9.19
Cases	Observations	sd	171916	2.10
Cases	Observations	var	29555122130	4.42
Deaths	Observations	mean	220	3.89
Deaths	Observations	sd	435	1.96
Deaths	Observations	var	189051	3.83
Cases	WIS	mean	15840	0.27
Cases	WIS	sd	53117	0.28
Deaths	WIS	mean	31	0.23
Deaths	WIS	sd	65	0.28
