# Supplementary material for: Scoring epidemiological forecasts on transformed scales
Source: PLoS Comput Biol. 2023 Aug 29;19(8):e1011393. doi: 10.1371/journal.pcbi.1011393 (PMC10495027; doi:10.1371/journal.pcbi.1011393)
Supplement: S2 Table — Any forecast that met one of the listed criteria (represented by a row in the table), was removed. Those forecasts were removed in order to be better able to illustrate the effects of the log-transformation on scores and eliminating distortions caused by outlier forecasters. When evaluating models against each other (rather than illustrating the effect of a transformation), one would prefer not to condition on the outcome when deciding whether a forecast should be taken into account. (PDF) [file pcbi.1011393.s003.pdf]

True value	&	Median prediction
> 0		$> 100\times$ true value
> 10		$> 20\times$ true value
> 50		$< 1/50\times$ true value
$= 0$		> 100
