# Supplementary figures and images for: Scoring epidemiological forecasts on transformed scales
Source: PLoS Comput Biol. 2023 Aug 29;19(8):e1011393. doi: 10.1371/journal.pcbi.1011393 (PMC10495027; doi:10.1371/journal.pcbi.1011393)

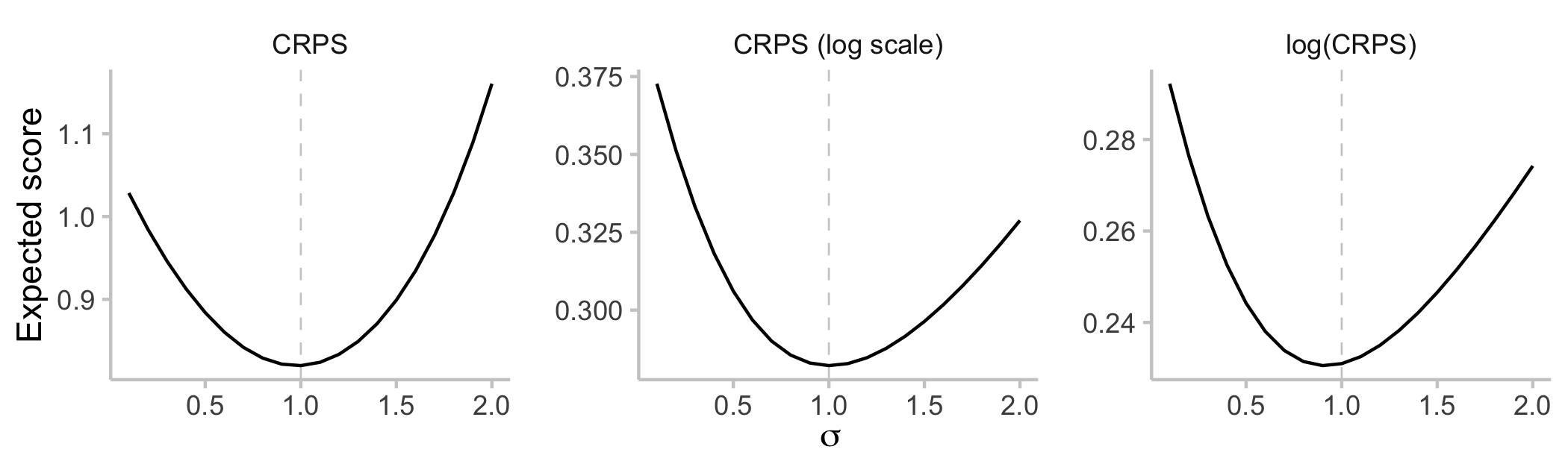

Supplement: S1 Fig — We assume Y ∼ LogNormal(0, 1) and evaluate the expected CRPS for predictive distributions LogNormal(0, σ) with varying values of σ ∈ [0.1, 2]. For the regular CRPS (left) and CRPS applied to log-transformed outcomes (middle), the lowest expectation is achieved for the true value σ = 1. For the log-transformed CRPS, the optimal value is 0.9, i.e. there is an incentive to report a forecast that is too sharp. The score is therefore no longer proper. (TIF) [file pcbi.1011393.s004.tif]

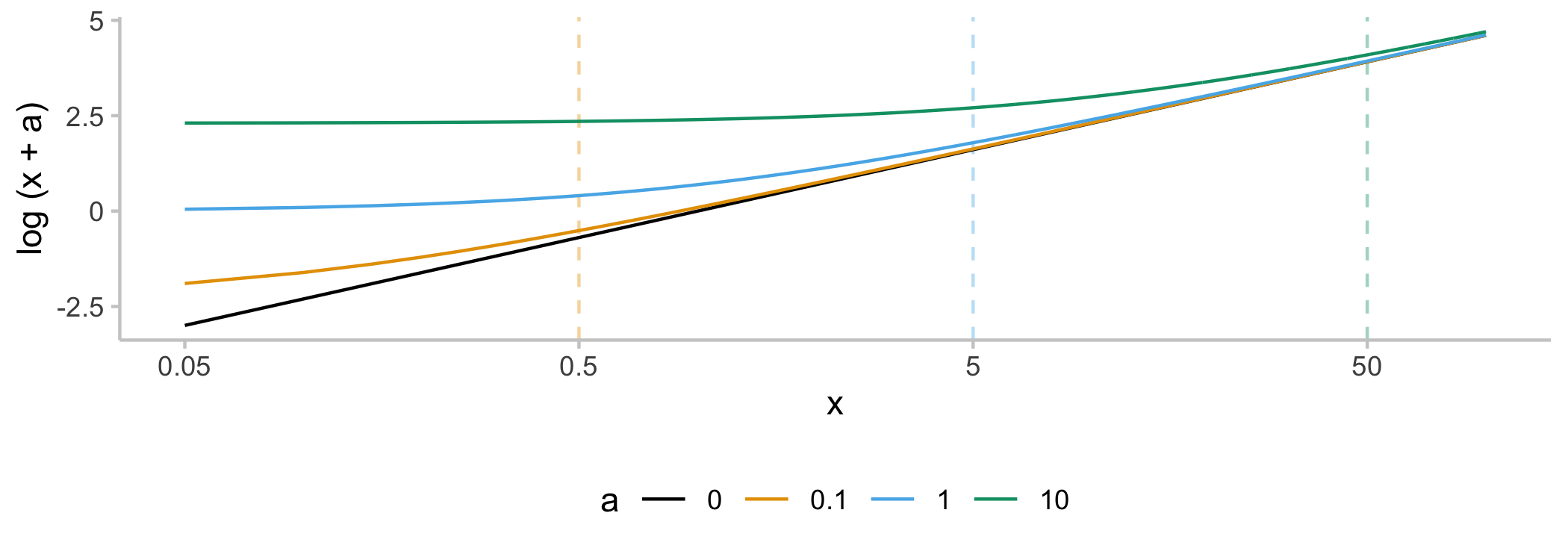

Supplement: S2 Fig — For increasing x, all lines eventually approach the black line (representing a transformation with no offset applied). For a given solid line, the dashed line of the same colour marks the x-value that is equal to 5 times the corresponding offset. It can be seen that for a values smaller than one fifth of the transformed quantity, the effect of adding an offset is generally small. When choosing a suitable a, the trade-off is between staying close to the interpretation of a pure log-transformation (choosing a small a) and not giving excessive weights to small observations (by choosing a larger a, see Fig 6). (TIF) [file pcbi.1011393.s005.tif]

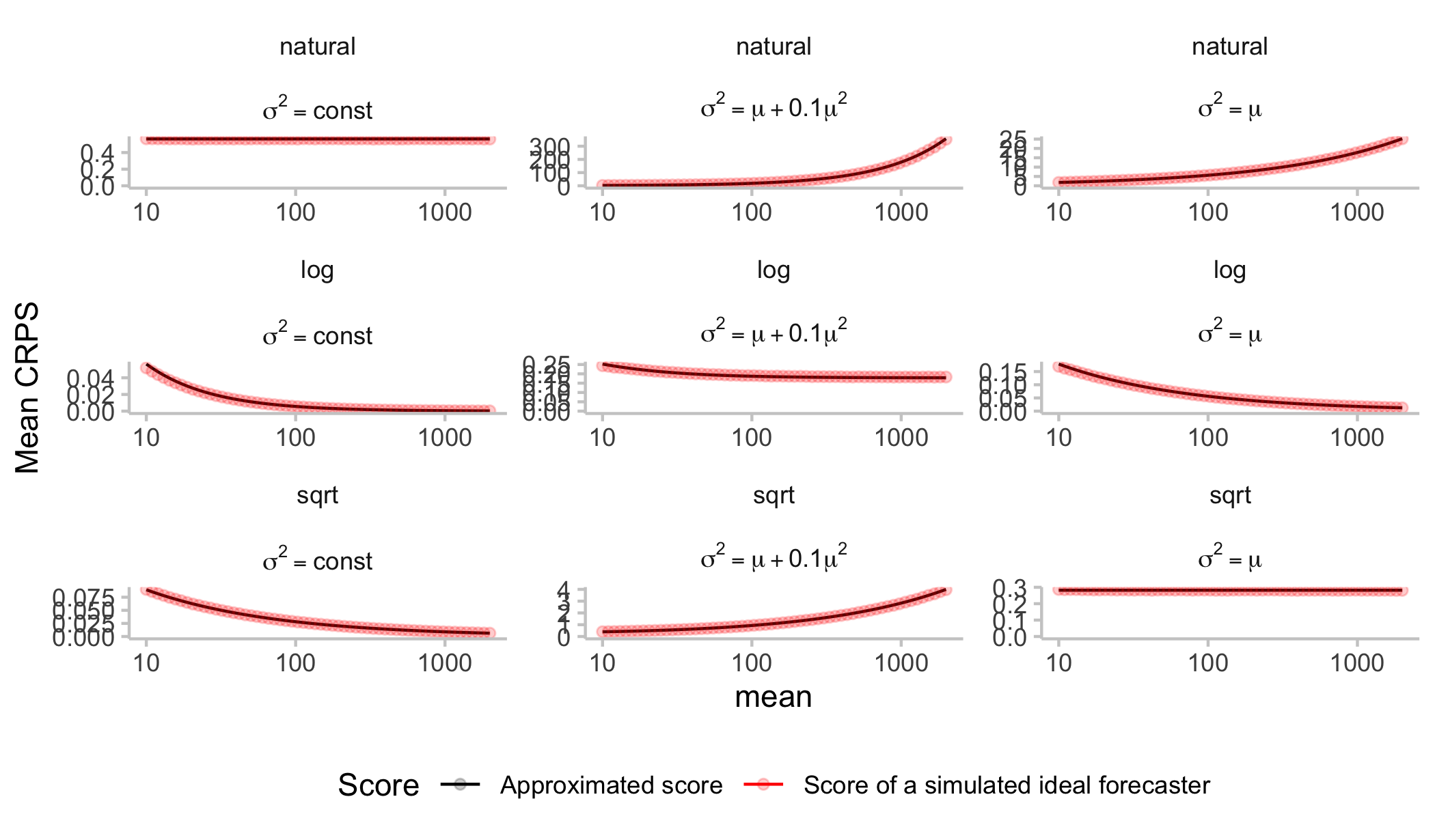

Supplement: S3 Fig — This is using the approximation detailed in theoretical discussion on model rankings (see also Fig 2). Expected CRPS scores are shown for three different distributions once on the natural scale (top row) and once scored on the log scale (bottom row). (TIF) [file pcbi.1011393.s006.tif]

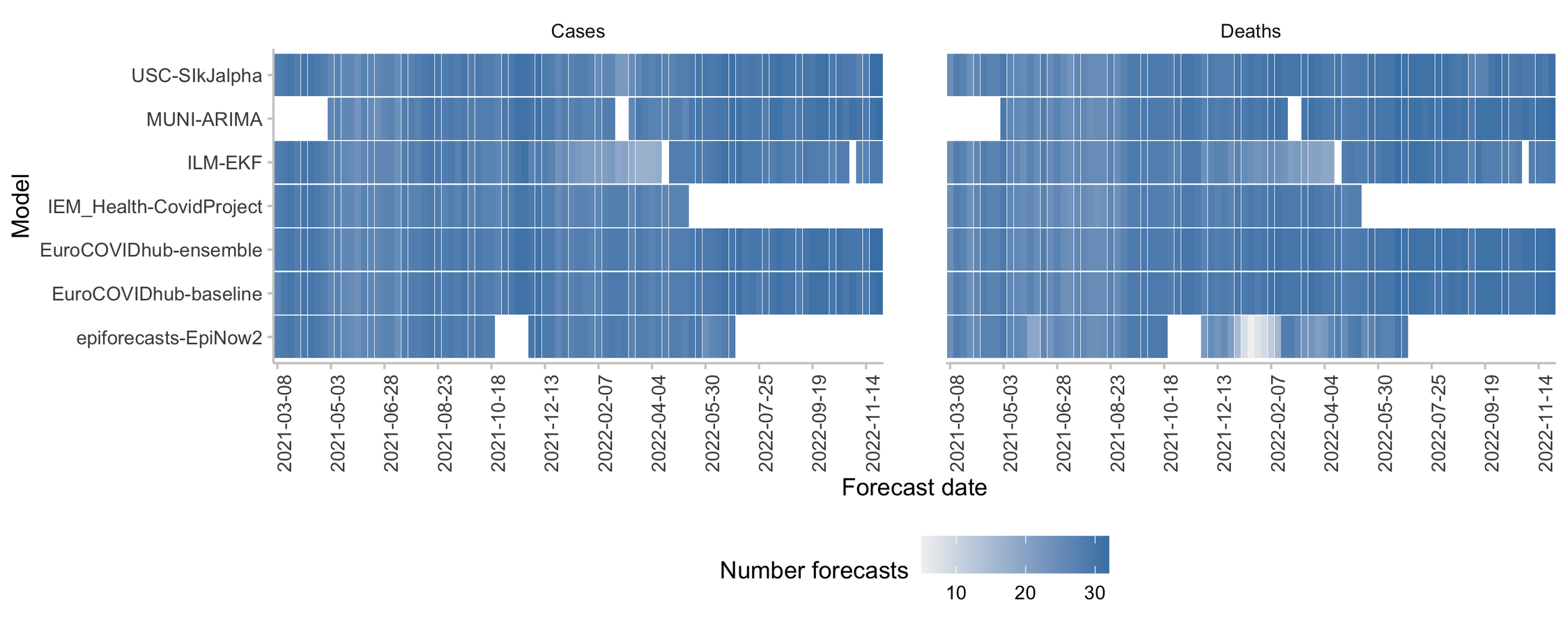

Supplement: S4 Fig — (TIF) [file pcbi.1011393.s007.tif]

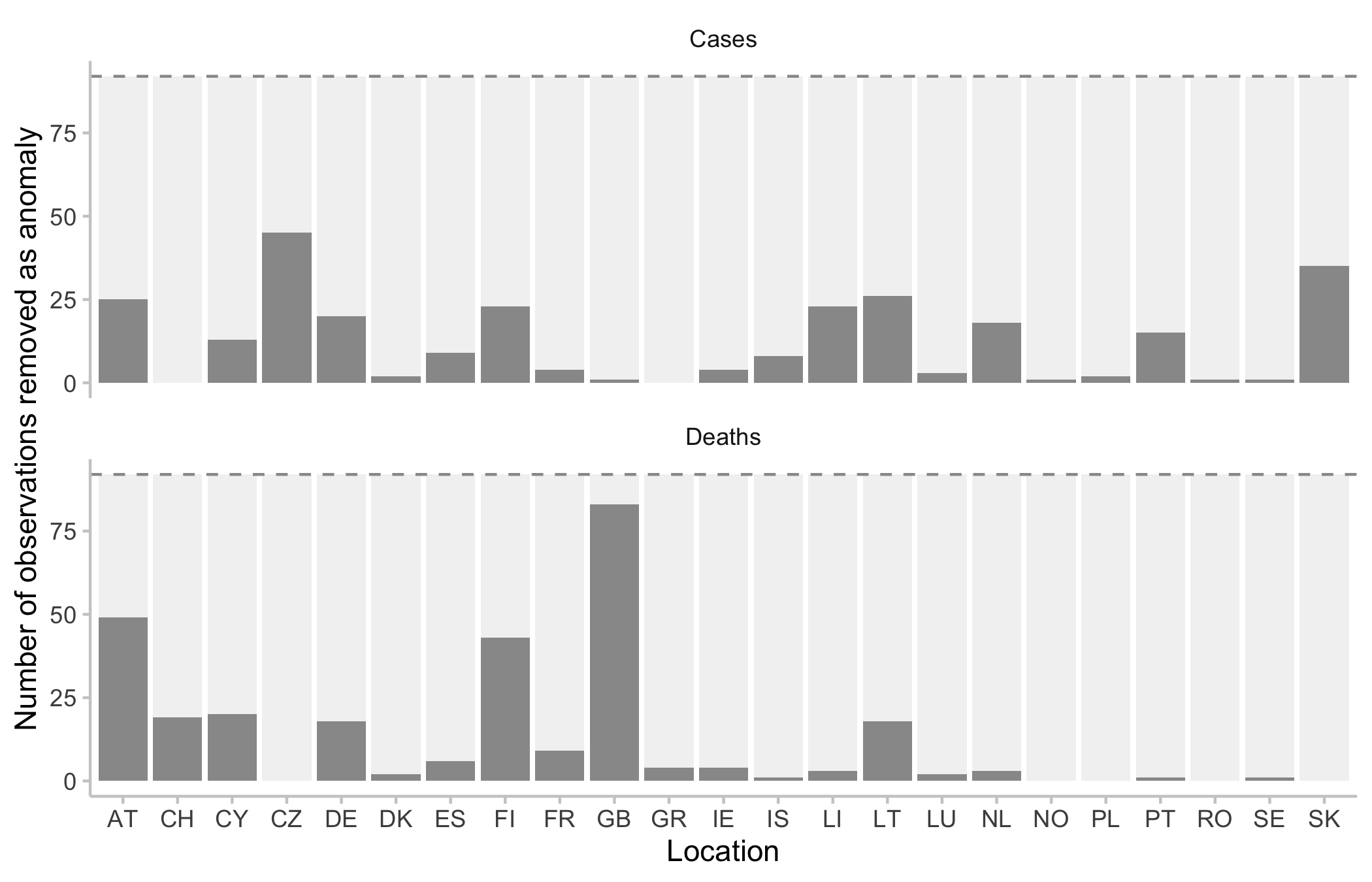

Supplement: S5 Fig — The values were marked as anomalous by the European Forecast Hub team. (TIF) [file pcbi.1011393.s008.tif]

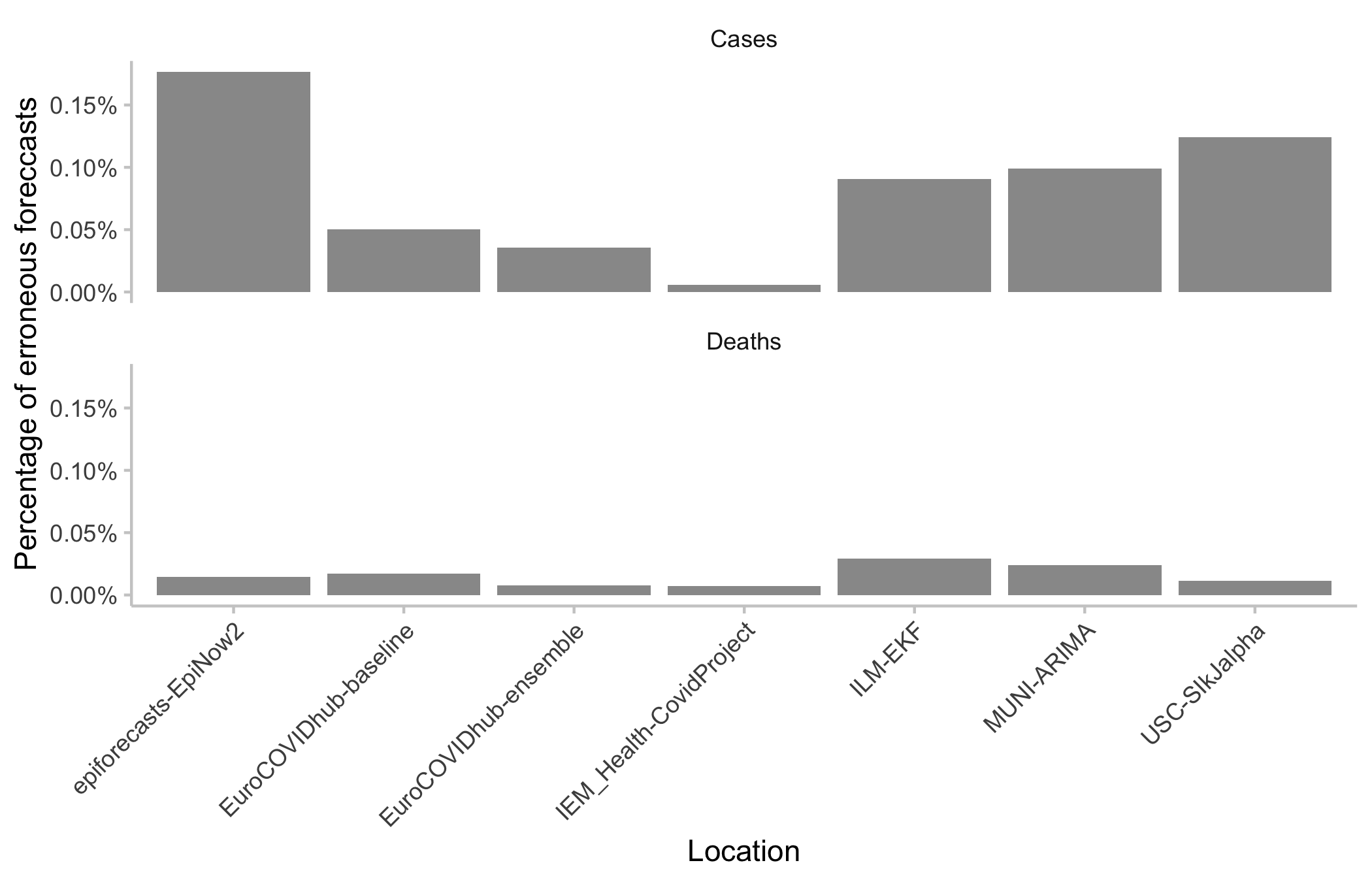

Supplement: S6 Fig — Forecasts that were in extremely poor agreement with the observed values were removed from the analysis according to the criteria shown in S2 Table. (TIF) [file pcbi.1011393.s009.tif]

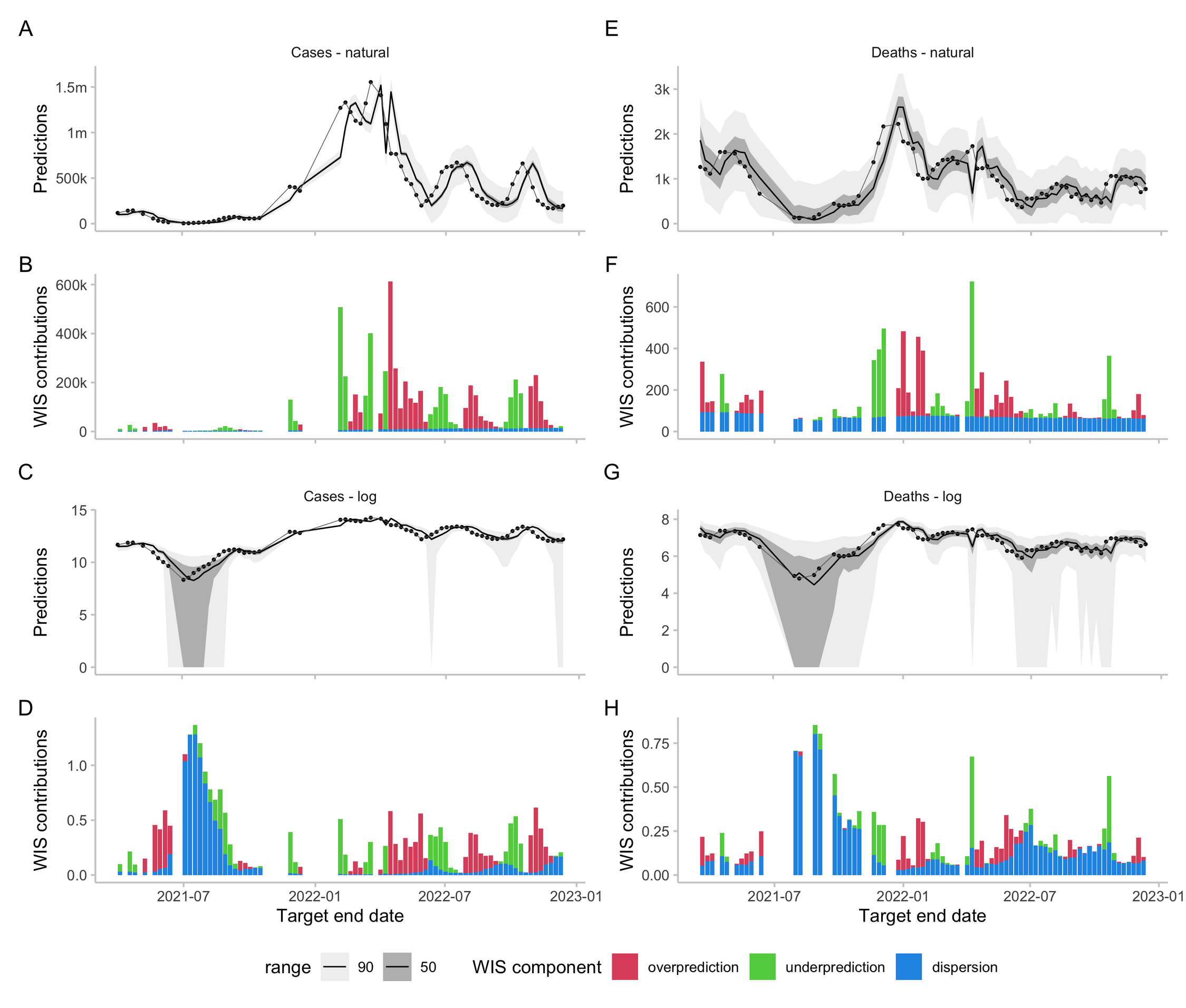

Supplement: S7 Fig — The model had zero included in some of its 50 percent intervals (e.g. for case forecasts in July 2021), leading to excessive dispersion values on the log scale. One could argue that including zero in the prediction intervals constituted an unreasonable forecast that was rightly penalised, but in general care has to be taken with small numbers. One potential way to do deal with this could be to use a higher a value when applying a transformation log(x + a), for example a = 10 instead of a = 1. A, E: 50% and 90% prediction intervals and observed values for cases and deaths on the natural scale. B, F: Corresponding scores. C, G: Forecasts and observations on the log scale. D, H: Corresponding scores. (TIF) [file pcbi.1011393.s010.tif]

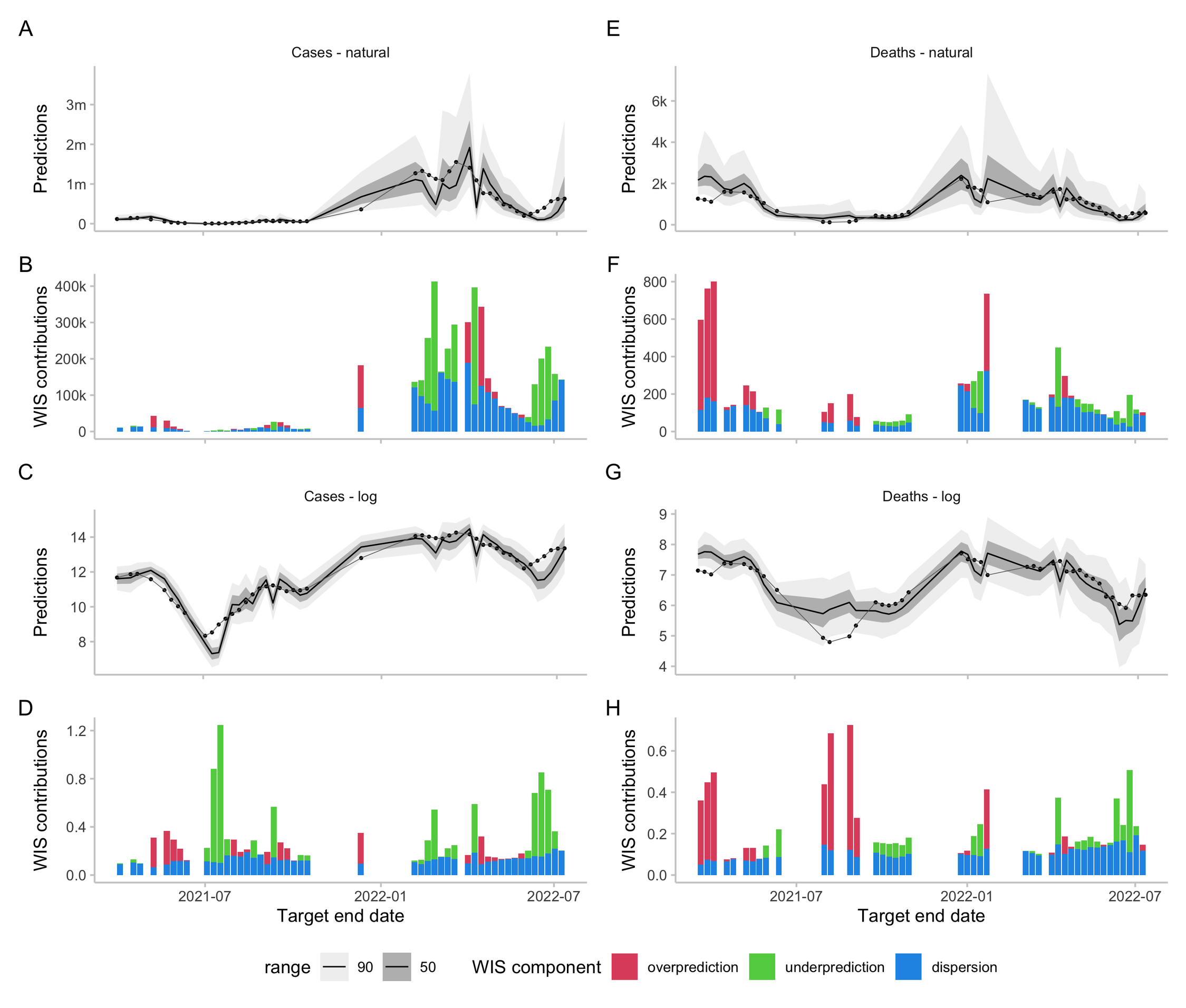

Supplement: S8 Fig — A, E: 50% and 90% prediction intervals and observed values for cases and deaths on the natural scale from the EpiNow2 model [38]. B, F: Corresponding scores. C, G: Forecasts and observations on the log scale. D, H: Corresponding scores. (TIF) [file pcbi.1011393.s011.tif]
