# Supplementary material for: Scoring epidemiological forecasts on transformed scales
Source: PLoS Comput Biol. 2023 Aug 29;19(8):e1011393. doi: 10.1371/journal.pcbi.1011393 (PMC10495027; doi:10.1371/journal.pcbi.1011393)
Supplement: Attachment — Submitted filename: comments.pdf [file pcbi.1011393.s012.pdf]

Summary

This manuscript develops theory, interpretations, and intuition regarding evaluation metrics applied to strictly monotonic transformations of observations and forecasts, with focus on log, shifted log, sqrt, and shifted sqrt transformations, and details the application and impact of using a log1p transformation within an evaluation framework for COVID-19 forecasts.

The analysis is extensive and covers many facets of selecting and applying such transformations, providing great value. However, it

- focuses on putting evaluations for different locations and times on an even scale, and does not directly answer the question of how to re-emphasize locations and times of import to forecast consumers, and
- the discussion of which locations and times forecasts consumers would want to emphasize seems incomplete.

The former point could be addressed by adding additional content regarding re-emphasis. The latter point could be addressed by studying what stakeholders want to emphasize and using that to make a recommendation for a particular transformation or transformation selection rule. Alternatively, both could be addressed simply by more explicitly and prominently noting that

- when selecting a transformation, one should consider the desired emphasis, which is likely not an even scale for most/all predictions, and
- the "natural logarithm" / log1p selection is not meant as a recommendation but to enable demonstration.

Additionally, any additional necessary/recommended elements of a scoring procedure should be noted more prominently (e.g., dealing with forecast missingness, outlying forecasts, outlying data, selecting a Box-Cox transformation, selecting a shift (a value), etc.).

Comments

Major

- "Natural logarithm", log1p, and $\log(a + x)$ are conflated in several places, despite the discussion in Section 2.4 noting that this is an important distinction. The terminology should not be overloaded. (E.g., L20 "Applying the log transformation".)
- L18:
  - "relative error" — Is this what stakeholders want? Or does it overemphasize low steady periods too much?
  - "under the assumption of quadratic mean-variance relationship" — And do we expect/observe this sort of relationship in epidemiological surveillance data? This reads more like an arbitrary assumption that will be taken instead of something that is examined in the

manuscript. (From Section 3.3 it seems like the answer is "roughly but not entirely".)
  - These points might be more simply resolved simply by revising L15 "motivate".
- L150: "it may be desirable" — Is it though? This seems unlikely. Stakeholders likely want to emphasize some locations and times over others, and would benefit from some rules about how to do so while maintaining propriety. (E.g., is scaling by some function of recent observations or the population size okay? Is scaling by the forecast or observed value okay? Or are we limited to monotonic transformations?)
- L166: "a negative binomial distribution with size parameter θ" — Is this realistic? If so, what's the interpretation epidemiologically?
- Figure 3: is a geometric distribution realistic for epidemiological surveillance data?
- L236: "better able to illustrate the effects" — Why does this removal help illustrate the effects? Is such a removal procedure required to be able to apply this transformation approach at all? And if so, what are the requirements/considerations for such a removal procedure?
- L244: "pairwise comparisons" — The necessity of using an approach to handle missingness when combining evaluations should be noted more prominently from the beginning. The same applies to the outlier removal (if necessary) and a value selection.
- Figure 4: The data (not) selected seems to obscure the key period of predicting the start of a case wave, as we expect the CRPS of log data to emphasize missed case increase predictions there more heavily than the natural scale. Deaths are not a substitute because they have access to a leading indicator and the same sort of misses may not be observed. The evaluations here may also miss some overshooting of the case peak. Which rules from Table SI.2 are coming into play and why? Is there a more complete alternative example?
- Table 1: The beta for cases and beta for deaths are both around 0.86 individually. But fit together they beta is 0.963; this appears due to trying to fit two different (difficulties of) tasks together that follow different trends, evident from the differing alpha values. A joint fit does not seem appropriate without allowing for a separate alpha per task type. The same thing can be said for the different horizons within cases&deaths. The fits here seem to better support an argument for something between sd and log.
- L279: "higher scores for smaller forecast targets" — This seems undesirable, given argument of Bracher et al. 2021a. Perhaps some forecast consumers would be interested in quality of forecasts for locations with lower activity (e.g., policymakers for smaller populations), but very unlikely would they want to prioritize less-active times in general over more-active times.

Minor

- L12 “over space and time” — Which do stakeholders care most about?
- L15 “log-transformed counts” — Immediately sounds problematic due to the possibility of 0 counts, without mention of a shift or threshold. Additionally, there is a truncated normal distribution in the mix; is it rounded to counts, or are these not all count data?
- L23–L24 “more strongly emphasized”, “less severely penalized” — Could note that this is relative to evaluations on the natural scale (not relative to each other).
- L24 “is only one” — Seems a bit too similar to “the only one”, “the only”, etc.
- L46 “WIS is an approximation of the CRPS” — Is it an approximation of CRPS itself or some (2x?) scaling?
- L59 “strength” — The meaning of “strength” is nonintuitive without reading the citation and understanding the ties to multiplicative interventions on R and r (the latter of which seems unrealistic to achieve). Consider surrounding with quotes, explaining more/less, relating to growth per generation, etc.
- L60: “immunity” $\rightarrow$ “population immunity”?
- L69: This might benefit from a short justification (e.g., $\frac{\left|x_t e^{(r_t+\epsilon)\Delta t} - x_t^{r_t \Delta t}\right|}{\left|x_t e^{(r_t-\epsilon)\Delta t} - x_t^{r_t \Delta t}\right|} = e^{\epsilon \Delta t}$)
- L70: Clarify: is this suggesting to make the forecasting targets themselves the result of applying some standardized growth rate estimator to current+future observations around the desired time period?
- L99: How are atoms at $\exp x = 0$ handled? How are observations of $y = 0$ handled?
- L127: “approximation of the absolute percentage error” — Raises a few questions:
  - If a relative error metric is desired, why not just divide CRPS/WIS by y then? (Propriety?)
  - How does using a (shifted) log transform differ from dividing by (a constant plus) (a mean of) the most recent observation(s)?
  - Is this desired? Though already discussed, seems like the major point of doubt here: are we at risk of overemphasizing very-low-activity periods and very-low-population states where standard-deviation-to-mean ratios are much higher?
- L127: As APE, RE, and SAPE are defined differently in other sources (e.g., with scaling factor in APE and SAPE, and with y in the denominator and no absolute value in RE), it may be helpful to also indicate sources for all three (e.g., that the RE definition is also in Gneiting, 2011).
- L138: “$\bar{r}$ is” — Consider $\bar{r}_t$ etc.
- L146: “$\bar{\hat{r}}$” — Should be $\hat{\bar{r}}$, or consider $\hat{\bar{r}}_t$.
- L158 “delta method” — Delta method would seem to also require the distribution to be somewhat tightly distributed, concentrated within a

nearly-linear region of the transformation function. But putting significant mass on a wider range of values would also mean having non-negligible mass on negative values if "approximately normal"; it would not make sense to apply the logarithm or sqrt transformations in these cases. So perhaps the "tightly distributed" assumption can be taken. But this seems a bit nonobvious and a justification/application of the delta method would be helpful here.

- L159 — Need to discuss / spell out the point of the above calculation (— that it is the same for all μ, when paired with the same c?).
- L168: "grows with the variance" — May read like this a linear growth. Consider "grows with the standard deviation".
- L180: "data-generating" — Consider "fitted"/"believed".
- L186: "incentivizes overconfident predictions" — Needs justification/citation/removal. This single example disproves propriety but doesn't establish overconfidence as being encouraged in general.
- Figure 2: Were any zeros encountered for the negative binomial or Poisson distributions? Was a shifted log transformation used rather than an unshifted log transformation?
- L215: "is mainly driven" — Please add pointer to justification (e.g., Figure 7 / discussion).
- L231: "only include models which" — Consider "only include the 7 models that"
- Figure 5 caption:
  - "logarithmic" — With what a values? (THe first link to Figure 5 is from before this information was stated.)
  - "D: [. . .] all individual scores" — of EuroCOVIDhub-ensemble or any of the 7 included systems?
  - "relative change" — of what change in scores relative to what quantity?
- L272: "with the standard deviation" — Also note here that this is for approximately normal data distributions?
- L282: "independent" — Consider "linearly independent".
- L288–290: What would stakeholders want this difference to be, and in which direction? I would guess the sqrt would generally be preferable unless applying some extra transformation after the log to re-emphasize locations/times of interest.
- Figure 7 caption: "one score" — Consider clarifying that/whether there is potentially missingness here.
- Figure 8: What is the meaning of the arrow colors? Initially it seems to match the direction, but there is also blue arrow pointing downward. If it is indicating the direction, please fix this and consider a third color for indicating unchanged ranks.
- The WIS contribution decomposition suggests that an alternative baseline should be used based on differences in transformed data rather than differences in natural scale data. Or, alternatively, that the a value is too low to give reasonable comparisons (as data that is hard/impossible to distinguish from 0 on a plot on the natural scale corresponds with very

high penalization of forecasts considering 0 as a possibility). This could (also) be noted in Figure SI.5's caption.

- L340: "convert [...] rate" — Please clarify. Does "multiplicative growth rate" refer to a setup with $\mathbb{E}[X_{t+k} \mid X_t] = X_t\theta^k$, or only a single step $\mathbb{E}[X_{t+\text{one step}} \mid X_t] = X_t\theta$? Does the proposed conversion apply to both, or only to the latter?
- L341–342: "natural logarithm as a [...] (VST)" — for what type of relevant distribution(s)?
- L344: "take differences between forecasts on the log scale" — Clarify.
- L345: "divide each forecast by the forecast" — Caveat about current forecast formats should be included here / in following sentences, without (one mention of) the idea about directly soliciting forecasts of week-on-week ratios in between.
- L366: "[...] settings" — (to standardize between measurements, then could potentially scale by importance to stakeholders)
- Figure SI.2: Would benefit from some more discussion, and perhaps a plot of slightly different quantities. E.g., to select an appropriate value of a, it seems like a common goal would be to deprioritize errors around values of x low enough that we expect there to be primarily noise rather than signal or that are not of concern to policymakers. For count data, this likely includes the values $0 - -10$ in any circumstances; how far beyond 10 this range extends depends on the range of realistic growth rates, the forecast horizon, and cleanliness of the data. To assist in this decision, it seems like a similar plot of the evaluations of a particular absolute and/or relative error might be useful, or discussion of how to read it from the current plot. E.g., to illustrate that the same relative error is penalized roughly the same for all the plotted values of a for values of x above 50, but quite differently for $a = 10$ vs. $a = 0.1$ for values of x near 1.
- Figure SI.7:
  - Also specify sorting variable&direction on x axis. (Would a point plot vs. the sorting variable be more informative?)
  - Very informative figure, promote to main text? Also would like to see for different "parts" of an epidemic (e.g., steady low periods, increases, peaks/plateaus, decreases). Deaths figures suggest that balancing scores between locations can be tricky, and both cases and deaths suggests that the investigated transformations can't do this exactly.

Typographical

- L96: "transformtation" → "transformation"
- Figure 2 caption: "CRPS values where computed" → "[...] were computed"
- L192: "does however influences" → "does however influence"
- In several places, e.g., L201, there are links to SI material, when clicked, that instead redirect to main text figures.
- L249: use two backticks on the left and two single quotes on the right in

LaTeX code for the quoting here

- Figure 5: “Loaction” $\rightarrow$ “Location”
- Figure SI.7: “Loaction” $\rightarrow$ “Location”
