# Supplementary material for: Scoring epidemiological forecasts on transformed scales
Source: PLoS Comput Biol. 2023 Aug 29;19(8):e1011393. doi: 10.1371/journal.pcbi.1011393 (PMC10495027; doi:10.1371/journal.pcbi.1011393)
Supplement: Attachment — Submitted filename: reply_to_reviews.pdf [file pcbi.1011393.s013.pdf]

We thank the reviewers for their feedback and thoughtful comments which we feel have helped improve the manuscript substantially. Please see our responses to the individual comments as well as explanations on the changes we have made to the manuscript below. In addition to the changes outlined below we made some general edits to the paper. We changed the title to make it more concise, we altered the order of some sections and shortened the introduction a bit, which had after the revision had become somewhat long.

1 Reviewer #1

Bosse et al. discuss the important problem of forecast evaluation in the context of epidemiological models. They make a clear and compelling argument for the use of different variance-stabilising transformations to reduce the impact of variation in model outputs and data over several orders of magnitude, a consequence of the exponential nature of epidemic growth (with time-varying growth rate).

I only really have minor, hopefully constructive points to raise — overall I think this is a very nice piece of work that deserves to be published in close to its current form. My big picture and lower level comments and suggestions follow.

Thank you for your kind words.

1.1 Big picture

1.1.1 Proper scoring rules

As noted by the authors, proper scoring rules are a commonly used concept in evaluating probabilistic predictions. While probably familiar to a reasonable fraction of readers, I think there is also likely a reasonable set of readers who are not aware of these or are only vaguely aware of them. For this reason I think a little more general background would help. For example on line 38 after the 'report their true belief about nature' another sentence or two along the lines of

"For example, some methods of 'scoring' probabilistic predictions can be 'gamed' in the sense that forecasters can do better by reporting a probability distribution different to their best estimate. A proper scoring rule ensures that the expected score when reporting a distribution Q, as evaluated under their actual best estimate of the probability distribution P, is best when Q = P. Thus a proper scoring rule encourages a forecaster to provide 'honest' predictions."

In addition, I suspect there are a few readers (such as myself!) who are more familiar with more 'basic' scores such as e.g. logarithmic score or arise (i.e. essentially the likelihood or related quantities). I spent a bit of time wondering about why these weren't considered, especially as these avoid the scale issues at the centre of the present article. Such scores were only briefly considered in the discussion, in the second-to-last paragraph. I think it would be helpful to mention/emphasise up front some of these 'classic' scores that don't have the same issues with scaling and then discuss why they aren't used, e.g. due to 'robustness' or other issues (e.g. lack of full probability distributions from forecasts).

We updated the paragraph, which now reads:

> Proper scoring rules are constructed such that they encourage honest forecasting and cannot be 'gamed' or 'cheated'. Assuming that the forecaster's actual best judgement corresponds to a predictive distribution F, a proper score is constructed such that if F was the data-generating process, no other distribution G would yield a better expected score. A scoring rule is called *strictly* proper if there is

no other distribution that under F achieves the *same* expected score as F, meaning that any deviation from F leads to a worsening of expected scores. Forecasters (anyone or anything that issues a forecast) are thus incentivised to report their true belief F about the future. Common proper scoring rules are the logarithmic or log score (Good, 1952) and the continuous ranked probability score (CRPS, Gneiting and Raftery, 2007). The log score is the predictive log density or probability mass evaluated at the observed value. It is supported by the likelihood principle (Winkler, 1996) and has many desirable theoretical properties; however, the particularly severe penalties it assigns to occasional misguided forecasts make it little robust (Bracher et al., 2021). Moreover, it is not easily applied to forecasts reported as samples or quantiles, as used in many recent disease forecasting efforts. It is nonetheless occasionally used in epidemiology (see e.g., Held et al. 2017; Johansson et al. 2019), but in recent years the CRPS and the weighted interval score (WIS, Bracher et al., 2021) have become increasingly popular.

1.1.2 Log vs other transformations

The authors take the position that the log transformation is generally preferable for their use case, though they discuss and compare others. Relatedly, their arguments in terms of relative error also involve an essentially log-linear (i.e. log data + additive error) form which may be pragmatic but somewhat inelegant imo.

In the theoretical part of the manuscript we simplify the equations by looking at the case of a single point forecast, rather than a probabilistic forecast that comes in the form of a predictive distribution. We feel this makes the discussion more intuitive. We want to emphasise that the log-linear error ε^* is really the error that is measured by the score, not the error model that would be assumed by any forecaster making a prediction.

As the authors note, a particularly controversial issue for the log transformation is the issue of zero values. They take the standard pragmatic stance that we can use log (eps + y) instead of log y in such cases. Many communities e.g. econometrics are vehemently opposed to this, preferring e.g. quasi-Poisson regression and robust standard errors in the context of estimation. While not completely opposed to log (eps + y) myself (I have used it too!) it makes me a bit uncomfortable I still can't help but feel that something along the lines of e.g. logarithmic/quasi-likelihood or deviance scores could be formulated that would be preferable to the log data transform. This would offer both automatic scaling and handling of zero values, in principle. However, I haven't thought carefully enough to offer a concrete alternative and the log(eps + y) approach appears to work reasonably here. Instead, and in combination with the previous point, perhaps a bit more discussion of the potential alternatives based on quasi-likelihood-style functions rather than data transformations could be added?

Thank you for raising this interesting point. The log score is indeed an alternative which is scale-invariant under strictly monotonic transformations. We now mention this in the introduction and state that for the log score the question of which scale to evaluate forecasts on does not arise. In our understanding, a quasi-likelihood approach (as opposed to an actual likelihood approach as in the log score) may not actually be necessary as at least in theory full predictive distributions are to be evaluated. Developing further ideas based on some form of quasi-likelihood would likely require more in-depth investigation, which we feel is beyond the scope of the current paper.

1.1.3 Observation and process models

The interpretation in e.g. 2.2 appears to essentially assume a deterministic process model and additive error on the log scale (multiplicative on the natural scale). Although more of a motivating heuristic than strict

assumption, a deterministic process model based on a mean will not in general be the same as a stochastic process model, right? Perhaps a further caveat that this is a fairly simplistic motivating tool might be useful?

Furthermore, why not assume that the model mean (say the output of the deterministic model) defines e.g. the mean of something like a Poisson distribution (probably in overdispersed/quasi form)? or negative binomial? The potential use of these distributions is considered in later sections in the context of motivating variance transforms, but then would again seem to motivate a (quasi-)likelihood-style score beyond the approximate transforms (though with pros and cons in terms of robustness, applicability in the presence of partial information).

It is important to note that the equations in 2.2 do not refer to any particular model used by a forecaster or any model that was fit to data. Rather, we define the instantaneous growth rate for a single realised epidemic and then show that scoring forecasts for that on the log scale equates to scoring a forecast of the average growth rate according to this definition (this is essentially the same argument as in the first comment of Section 1.1.2 above). The forecaster themselves who would make a forecast could assume any error model or model structure they like.

1.2 Specific suggestions

I realised as I was about comment on some equations that none of the equations are numbered. It would probably be good to number them :-)

You are completely right, we added numbers to all equations.

The first equation (line 43) uses an unbolded indicator function which isn't defined (this also appears in the same form in the third equation, line 99). The second equation in contrast uses bold and defines the indicator. The definition should be moved forward and a choice of bold or not made.

We added the definition to equation according to your suggestion 1 and used bold throughout the manuscript consistently.

I think the term 'propriety' (as in having the property of being proper) should either be explicitly defined or re-worded in terms of 'being proper'.

We reworded all occurrences of the term 'propriety' in the text.

No reference is provided for the approximate variance-stabilising properties of the square root transformation.

We added a reference to the following book: Dunn, P.K., Smyth, G.K.(2018). Generalized Linear Models With Examples in R. Germany: Springer New York, page 118.

2 Reviewer #2

Summary

This manuscript develops theory, interpretations, and intuition regarding evaluation metrics applied to strictly monotonic transformations of observations and forecasts, with focus on log, shifted log, sqrt, and shifted sqrt transformations, and details the application and impact of using a log1p transformation within an evaluation framework for COVID-19 forecasts. The analysis is extensive and covers many facets of selecting

and applying such transformations, providing great value.

However, it

- focuses on putting evaluations for different locations and times on an even scale, and does not directly answer the question of how to re-emphasize locations and times of import to forecast consumers, and
- the discussion of which locations and times forecasts consumers would want to emphasize seems incomplete.

The former point could be addressed by adding additional content regarding re-emphasis. The latter point could be addressed by studying what stakeholders want to emphasize and using that to make a recommendation for a particular transformation or transformation selection rule. Alternatively, both could be addressed simply by more explicitly and prominently noting that

- when selecting a transformation, one should consider the desired emphasis, which is likely not an even scale for most/all predictions, and
- the "natural logarithm" / log1p selection is not meant as a recommendation but to enable demonstration.

Thank you for your thorough and helpful comments.

We added the following to the discussion to make our intentions clearer regarding the preferences of policy makers:

> In this paper, we did not make any particular assumptions about policy makers' priorities and preferences. Rather, we aimed to enable users to make an informed choice by showing how different transformations lead to different relative weights for the kinds of prediction errors forecast consumers may care about, such as absolute vs. relative errors or the size of penalties for over- vs. underprediction. In practice, engagement with decision makers is important to determine what their priorities are and how different ways to measure predictive importance should be weighed.

It is important to note that changing the relative weight for different times and locations is one effect of the scoring forecasts on the log scale, but not necessarily the main point of the transformation. Rather, we do recommend to evaluate forecasts on the log scale as we believe the interpretation as a score of the exponential growth rate is meaningful in an epidemiological context. As stated explicitly in the manuscript, we moreover recommend to use this in complement to rather than as a replacement of the common evaluation on the "natural scale". Evaluators will then become aware of whether their results depend strongly on this decision and the implied weighting of different forecasting tasks.

Users who are explicitly concerned about the relative weights of different times and locations can e.g. find information in Figure 3 where we show how different forecast targets are emphasized by evaluation on the natural or log-transformed scale. A user could e.g. reproduce such a display with different values of the offset a and choose a such that the relative weighting of times and regions with high and low incidence correspond to their preferences.

Additionally, any additional necessary/recommended elements of a scoring procedure should be noted more prominently (e.g., dealing with forecast missingness, outlying forecasts, outlying data, selecting a Box-Cox transformation, selecting a shift (a value), etc.).

To direct the readers' attention to the mentioned challenges already in the conceptual part of the paper we added the following paragraph to Section 2.4, which also contains a discussion of the choice of a:

> We note that in the practical evaluation of operational forecasting systems several additional challenges arise, which we do not study in detail. These concern e.g., the removal of outlying observations and forecasts and the handling of missing forecasts. The solutions we employed in practice are provided in Section 3.1.

We added a paragraph on which kinds of transformations are permissible in Section 4 (see comments below). We agree that it would be useful to have a more thorough discussion of all the considerations involved in evaluating forecasts, but feel this is slightly beyond the scope of this paper.

2.1 Major comments

"Natural logarithm", log1p, and $\log(a + x)$ are conflated in several places, despite the discussion in Section 2.4 noting that this is an important distinction. The terminology should not be overloaded. (E.g., L20 "Applying the log transformation".)

We changed "Applying the log transformation" to "Applying a transformation of $\log(x + 1)$" in L20 (in the abstract).

We slightly disagree with the notion that the terminology should not be overloaded. Not introducing another term for transformations of $\log_e(x + \varepsilon)$ makes the manuscript more readable and accessible to readers. In addition, our analyses suggest that adding a small offset does not make much of a difference in most practical applications. We nevertheless completely agree that this should be made clear to the reader and have added the following text:

> For conceptual clarity and to allow for a more in-depth discussion, we focus mostly on the natural logarithm as a particularly attractive transformation in the context of epidemic phenomena. We refer to this transformation as 'log-transformation' and to scores that have been computed from log-transformed forecasts and observations as scores 'on the log scale' (as opposed to scores 'on the natural scale', which involve no transformation). In the theoretical discussion in Section 2, 'log-transformation' and 'log scale' generally refer to a transformation of $\log_e(x)$. For practical applications (Section 3) we also use these terms to describe a transformation of $\log_e(x + a)$ with a small $a > 0$ in order to keep the terminology and notation simple. For a prediction target with strictly positive support, the CRPS after applying a log-transformation is given by

L18:

- "relative error" — Is this what stakeholders want? Or does it overemphasize low steady periods too much?
- "under the assumption of quadratic mean-variance relationship" — And do we expect/observe this sort of relationship in epidemiological surveillance data? This reads more like an arbitrary assumption that will be taken instead of something that is examined in the manuscript. (From Section 3.3 it seems like the answer is "roughly but not entirely".)
- These points might be more simply resolved simply by revising L15 "motivate".

We revised the word "motivate" in line 15. The sentence in the abstract now reads:

> Using the CRPS on log-transformed values as an example, we list three attractive properties: [...].

We think the relative error is one option out of many that could be interesting to policy makers. Paireau et al. (2022, `https://www.pnas.org/doi/pdf/10.1073/pnas.2103302119`) for example specifically emphasize

that in their work the use of MAPE is motivated by its straightforward interpretation. But we agree that ultimately stakeholders need to decide for themselves what metrics they are interested in. As mentioned above, adjusting the offset before taking the logarithm is one way of dealing with periods of low counts, but this of course moves the interpretation of the score away from a score of the exponential growth rate.

The assumption of a quadratic mean-variance relationship is not entirely arbitrary, but rather has a specific interpretation in the sense that it encodes the assumption that one can forecast the growth rate equally well regardless of the observed magnitude at the moment. In the manuscript, we state:

> The assumption of a quadratic mean-variance relationship is closely linked to the aspects discussed in Sections 2.1 and 2.2. It implies that relative errors have constant variance and can thus be meaningfully compared across different targets. Also, it arises naturally if we assume that our capacity to predict the epidemic growth rate does not depend on the expected outcome, i.e. does not depend on the current phase of the epidemic or the order of magnitude of current observations.

For a specific motivation in a simple branching process model please see our reply to comment "L166" below.

L150: "it may be desirable" — Is it though? This seems unlikely. Stakeholders likely want to emphasize some locations and times over others, and would benefit from some rules about how to do so while maintaining propriety. (E.g., is scaling by some function of recent observations or the population size okay? Is scaling by the forecast or observed value okay? Or are we limited to monotonic transformations?)

We think that while it is certainly not the only relevant approach, there is a case to be made for an evaluation which gives each forecasting target a similar weight. This is attractive if the goal of the evaluation is to find the best forecasting model for future use and if one assumes that the difficulty of the forecasting task is similar in different locations. Take for example two models that make independent forecasts for cases of COVID-19 in Germany, Luxembourg and Liechtenstein. CRPS scores on the natural scale would be dominated by the predictive performance in Germany. One model could be a lot better in Luxembourg and Liechtenstein and slightly worse in Germany and end up looking worse in terms of overall CRPS. However, it is unclear whether predictive performance in Germany really provides e.g. 100 times more information about how good a model is at predicting disease dynamics. If the question is which model to deploy in another location in the future, then it might be desirable to not have average scores be dominated by a single location.

We have updated the paragraph as such:

> When evaluating models across sets of forecasting tasks, it may be desirable for each target to have a similar impact on the overall results. This could be motivated by the assumption that forecasts from different geographical units and time periods provide similar amounts of information about how well a forecaster performs. One would then like the resulting scores to be independent of the order of magnitude of the target to predict. CRPS values on the natural scale, however, typically scale with the order of magnitude of the quantity to be predicted.

Regarding the question which transformations are permissible:

Scaling by past observations is permissible (indeed taking the log is similar to scaling by the last value), as is scaling by the population size as mentioned in the discussion. There may of course be exceptions such as dividing by zero, but otherwise this just represents a monotonic transformation, which is unproblematic in terms of propriety. Scaling a forecast by the later observed value (as opposed to scaling by past observations) is generally not permissible as it disturbs propriety; see Lerch et al (2017, Statistical Science, preprint: `https://arxiv.org/abs/1512.09244`) on the closely related topic of weighting scores with a function of the observed value. Similarly, scaling a forecast by predictions made by other forecasters may break propriety, but we are unaware of existing theoretical arguments. Scaling scores, e.g. dividing a score by the mean score for other forecasters may be acceptable in most applied settings, but we are unsure about exactly when and

how this could break propriety (as is the case for example when dividing by zero).

To make this clearer in the text, we added the following paragraphs:

> A natural question is which other transformations could be applied and whether resulting scores remain (strictly) proper. In principle, any transformation function can be applied simultaneously to forecasts and observations as long as the definition of the transformation is independent of the forecasts and any quantities unknown at the time of forecasting, including the observed value. This simply corresponds to a re-definition of the forecasting target. However, applying non-invertible transformations leads to a loss in information conveyed by forecasts, which we consider undesirable. The resulting score will be proper, but it may not be strictly proper anymore (as forecasts differing from the forecaster's true belief on the original scale may be identical on the transformed scale). When using the CRPS or the WIS, it seems most appropriate to use only strictly monotonic transformations such as the natural logarithm or the square root as otherwise the encoded notion of distance may become meaningless.
>
> Some other strictly monotonic transformations that can be applied are scaling by the population size or scaling by past observations. The latter, as discussed in Section 4, is similar to applying a log-transformation, but corresponds to evaluating a forecast of multiplicative, rather than exponential growth rates. The arising issue of dividing by zero can again be solved by adding a small offset a. Scaling a forecast by the later observed value (as opposed to scaling by past observations) is generally not permissible as it can result in improper scores (see Lerch et al. 2015 on the closely related topic of weighting scores with a function of the observed value). Similarly, scaling forecasts and observations by a function of the predictive distribution (like the predictive mean) may lead to improper scores; however, we are unaware of existing theoretical arguments on this.
>
> When applying a transformation, the order of the operations matters, and applying a transformation after scores have been computed generally does not guarantee that the score remains proper. In the case of log transforms, taking the logarithm of the CRPS values, rather than scoring the log-transformed forecasts and data, results in an improper score. We illustrate this point using simulated data in Figure SI.1, where it can be seen that in the example overconfident models perform best in terms of the log WIS. We note that strictly speaking, re-scaling average scores by the average score of a baseline model or average scores across different models to obtain skill scores likewise leads to improper scores (Gneiting and Raftery, 2007). The application of such skill scores, however, is established practice and considered largely unproblematic.

L166: "a negative binomial distribution with size parameter θ" — Is this realistic? If so, what's the interpretation epidemiologically?

This is an illustrative example and is not meant to be completely realistic. Rather, the point is to illustrate that for observations which have a standard deviation that grows linearly with the mean, the log transformation can serve as a variance-stabilising transformation. However, negative binomial distributions are commonly used in the epidmeiological literature; see e.g., Section 3 of Meyer and Held (2017, `http://dx.doi.org/10.1214/14-AOAS743`). An epidemiological interpretation could be the following (see e.g. Funk, Abbott and Bracher `https://doi.org/10.1111/rssa.12974`):

- For simplicity assume a generation time of one time interval (this could be relaxed).
- Each of the Y_{t-1} infected individuals at time $t-1$ generates new infections at time t, with a reproduction number of R_t and independent Poisson offspring distributions. Assuming independence across infecting individuals and given R_t and Y_{t-1}, Y_t then follows a Poisson distribution with mean $R_t \times Y_{t-1}$.
- We assume that R_t is not fixed, but given by

$$R_t = R_0 \times \theta_t$$

where

$$\theta \sim \text{Gamma}(\text{shape} = \psi, \text{rate} = \psi).$$

R_t thus fluctuates around an average value of R_0.

- This implies

$$Y_t \mid Y_{t-1} \sim \text{NegBin}(\text{mean} = R_t \times Y_{t-1}, \text{size} = \psi).$$

So a conditional negative binomial distribution with quadratic mean-variance relationship arises if we assume that a suitably distributed environmental factor θ_t modifies the reproductive number. While the assumption of this specific gamma distribution is arbitrary, a quadratic mean-variance relationship will also arise for other distributions of θ_t.

This illustrates that a quadratic mean-variance relationship can arise from a simple yet not completely unreasonable renewal equation model.

Figure 3: is a geometric distribution realistic for epidemiological surveillance data?

We agree that this is not a typical example we would expect to see in an epidemiological setting. We still believe the example is valuable to illustrate the general point that the rankings between two forecasts can change even for a single observation. We added the following sentence:

> We note that the chosen example involving a geometric forecast distribution is somewhat constructed; as shown in Section 3.4 and Figure 8A, rankings between models usually stay quite stable for a single forecast. .

L236: "better able to illustrate the effects" — Why does this removal help illustrate the effects? Is such a removal procedure required to be able to apply this transformation approach at all? And if so, what are the requirements/considerations for such a removal procedure?

We updated the paragraph as follows:

> In addition, we filtered out erroneous forecasts that were in extremely poor agreement with the observed data, as defined by any of the conditions listed in Table SI.2. Figure SI.6 shows the percentage of forecasts removed for each model. Those few (less than 0.2% of forecasts for each model) erroneous outlier forecasts had excessive influence on average scores and relative skill scores in a way that was not representative of normal model behaviour. We removed them here in order to better illustrate the effects of the log-transformation on scores that one would expect in a well-behaved scenario. In a regular forecast evaluation such erroneous forecasts should usually not be removed and would count towards overall model scores.

L244: "pairwise comparisons" — The necessity of using an approach to handle missingness when combining evaluations should be noted more prominently from the beginning. The same applies to the outlier removal (if necessary) and a value selection.

We added the following short paragraph to Section 2.4 to ensure these aspects are already mentioned in the conceptual part of the paper.

> We note that in the practical evaluation of operational forecasting systems several additional challenges arise, which we do not study in detail. These concern e.g., the removal of outlying observations and forecasts and the handling of missing forecasts. The solutions we employed in practice are provided in Section 3.1.

Figure 4: The data (not) selected seems to obscure the key period of predicting the start of a case wave, as we expect the CRPS of log data to emphasize missed case increase predictions there more heavily than

the natural scale. Deaths are not a substitute because they have access to a leading indicator and the same sort of misses may not be observed. The evaluations here may also miss some overshooting of the case peak. Which rules from Table SI.2 are coming into play and why? Is there a more complete alternative example?

The data that was removed here was largely due to data revisions, not due to forecasts removed by the filtering on our part. We added the percentages of observed values in the main text and added the following two Figures in the SI to make that clearer.

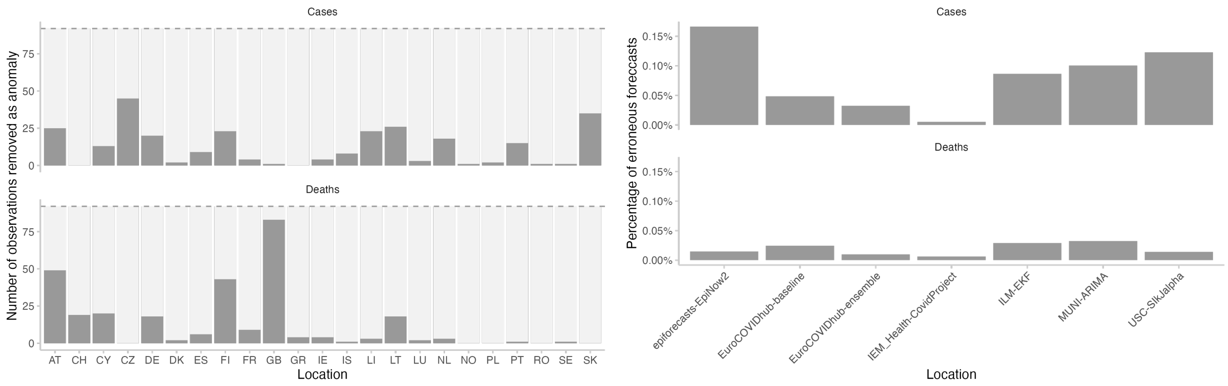

Figure 1:

Table 1: The beta for cases and beta for deaths are both around 0.86 individually. But fit together they beta is 0.963; this appears due to trying to fit two different (difficulties of) tasks together that follow different trends, evident from the differing alpha values. A joint fit does not seem appropriate without allowing for a separate alpha per task type. The same thing can be said for the different horizons within cases&deaths. The fits here seem to better support an argument for something between sd and log.

We agree with the point, thank you for raising it. We removed the joint fit from the table.

L279: "higher scores for smaller forecast targets" — This seems undesirable, given argument of Bracher et al. 2021a. Perhaps some forecast consumers would be interested in quality of forecasts for locations with lower activity (e.g., policymakers for smaller populations), but very unlikely would they want to prioritize less-active times in general over more-active times.

We generally agree that giving higher weight to targets with lower incidences may be undesirable in many instances (one important exception is instances where times of low incidences are followed by upswings). Empirically, in our example, the additional weight given to smaller forecast targets on the log scale was rather small compared to the excessive weight given to larger forecast targets on the natural scale. However we agree that this is a trade-off. One potential avenue for future exploration might be a composite score that combines scores on the natural and on the log scale.

2.2 Minor comments

L12 "over space and time" — Which do stakeholders care most about?

We think that this is entirely up to the stakeholders and may depend on the setting. We hope that the reasoning and trade-offs involved become sufficiently clear throughout the remainder of the paper.

L15 "log-transformed counts" — Immediately sounds problematic due to the possibility of 0 counts, without mention of a shift or threshold. Additionally, there is a truncated normal distribution in the mix; is it

rounded to counts, or are these not all count data?

We changed "log-transformed counts" to "log-transformed values" and updated the abstract to point out that we're applying a transformation of $\log(x+1)$ to our example data:

> Applying a transformation of log(x + 1) to data and forecasts from the European COVID-19 Forecast Hub

The truncated normal distribution used as an example in Figure 2 is based directly on samples from the normal distribution, so here no rounding was applied.

L23–L24 "more strongly emphasized", "less severely penalized" — Could note that this is relative to evaluations on the natural scale (not relative to each other).

Thank you for the suggestion. We updated the abstract as such:

> Situations in which models missed the beginning of upward swings are more strongly emphasised while failing to predict a downturn following a peak is less severely penalised when scoring transformed forecasts as opposed to untransformed ones.

L24 "is only one" — Seems a bit too similar to "the only one", "the only", etc.

We hope that the remainder makes it sufficiently clear that other transformations are possible.

L46 "WIS is an approximation of the CRPS" — Is it an approximation of CRPS itself or some (2x?) scaling?

The weighted interval score is a weighted sum of interval scores for the individual prediction intervals. When weighing the individual interval scores for the central $1-\alpha$ prediction intervals by $w=\alpha/2$ (which is generally standard), then the WIS converges exactly to the CRPS for an increasing number of equally spaced prediction intervals. For an illustration see Figure 2 in the referenced paper by Bracher, Ray, Gneiting and Reich (2021).

L59 "strength" — The meaning of "strength" is nonintuitive without reading the citation and understanding the ties to multiplicative interventions on R and r (the latter of which seems unrealistic to achieve). Consider surrounding with quotes, explaining more/less, relating to growth per generation, etc.

We revised and shortened the introduction, so this sentence has been removed. Now, it simply reads:

> Especially in their early phase, outbreaks are best conceived as exponential processes, characterized by potentially time varying reproduction numbers R_t (Gostic et al., 2020) or epidemic growth rates r_t (Dushoff and Park, 2021).

L60: "immunity" → "population immunity"?

We agree with you, but have simplified the introduction such that this sentence does no longer appear.

L69: This might benefit from a short justification (e.g., $\left|\frac{x_t e^{(r_t+\epsilon)\Delta t} - x_t e^{r_t \Delta t}}{x_t e^{(r_t-\epsilon)\Delta t} - x_t e^{r_t \Delta t}}\right| = e^{\epsilon \Delta t}$)

This is a great suggestion, thank you. We updated the corresponding paragraph, which now reads:

> If the true modelling task revolves around estimating and forecasting these quantities, then evaluating forecasts based on the absolute distance between forecasted and observed incidence values penalises underprediction (of the reproduction number or growth rate) less than overprediction by the same

amount. For illustration, consider an incidence forecast issued at time 0 and referring to time t that misses the correct average growth rate $\bar{r}_t$ by either $-\epsilon$ or $+\epsilon$. Then the ratio of the resulting absolute errors on the scale of observed incidences y_t is

$$\frac{|y_0 \exp[(\bar{r}_t - \epsilon) \times t] - y_0 \exp(\bar{r}_t t)|}{|y_0 \exp[(\bar{r}_t + \epsilon) \times t] - y_0 \exp(\bar{r}_t t)|} = \exp(-\epsilon t) < 1. \quad (1)$$

If one is to measure the ability to forecast the underlying infection dynamics, it may thus be more desirable to evaluate errors on the scale of the growth rate directly.

L70: Clarify: is this suggesting to make the forecasting targets themselves the result of applying some standardized growth rate estimator to current+future observations around the desired time period?

Here we aren't suggesting a specific transformation yet, but rather just say that it would be desirable to score a forecast of the growth rate (regardless of how that can be achieved). Specific suggestions for how to achieve this (either by taking a log transformation or by using another transformation to obtain growth rates directly as mentioned in the Discussion) are discussed in more detail in the remainder of the text.

L99: How are atoms at $\exp x = 0$ handled? How are observations of $y = 0$ handled?

In the theoretical discussion in Section 2 we defined prediction targets to have strictly positive support, so these cases are excluded. In the later parts we follow the common strategy to add an offset a before taking the logarithm.

L127: "approximation of the absolute percentage error" — Raises a few questions:

- If a relative error metric is desired, why not just divide CRPS/WIS by y then? (Propriety?)
  Yes, dividing by the observed value generally disturbs propriety. This approach would correspond to weighting CRPS scores with weights depending on the observed value, and it has been shown that the resulting average scores are no longer proper; see Lerch et al (2017, Statistical Science, preprint: `https://arxiv.org/abs/1512.09244`)
- How does using a (shifted) log transform differ from dividing by (a constant plus) (a mean of) the most recent observation(s)?
  Like in the related arguments in the paper we address this question for the simpler case of the absolute error rather than CRPS. Using a shifted log transform corresponds to (in slight variation of equation (8))

$$|\varepsilon_t^*| = |\log(\hat{y}_t + a) - \log(y_t + a)| = \left|\log\left(\frac{\hat{y}_t + a}{y_t + a}\right)\right| \overset{\text{if } \hat{y}_t \approx y_t}{\approx} \left|\frac{\hat{y}_t + a}{y_t + a} - 1\right| = \left|\frac{\hat{y}_t - y_t}{y_t + a}\right|. \quad (2)$$

Dividing by the last observation plus a constant a, on the other hand, corresponds to

$$|\varepsilon_t^*| = \left|\frac{\hat{y}_t + a}{y_{t-1} + a} - \frac{y_t + a}{y_{t-1} + a}\right| = \left|\frac{\hat{y}_{t-1} - y_t}{y_{t-1} + a}\right|. \quad (3)$$

If we interpret the denominator as an (inverse) weight for the different absolute errors, log transformation thus corresponds approximately equivalent to weighting with the inverse new observation y_t (though better behaved in terms of the resulting incentives). Dividing by the previous value corresponds to weighting with the inverse preceding observation. As autocorrelation is typically strong in the time series we consider, this will yield similar, but not identical results.
In terms of the epidemiological interpretation, dividing by the last observed value can be interpreted as a score for a multiplicative, rather than an exponential growth rate.

- Is this desired? Though already discussed, seems like the major point of doubt here: are we at risk of overemphasizing very-low-activity periods and very-low-population states where standard-deviation-to- mean ratios are much higher?
  We agree that it may be an issue in practice that low-activity-periods could get overemphasised and therefore believe that an evaluation should show both scores on the natural scale as well as on the log scale.

L127: As APE, RE, and SAPE are defined differently in other sources (e.g., with scaling factor in APE and SAPE, and with y in the denominator and no absolute value in RE), it may be helpful to also indicate sources for all three (e.g., that the RE definition is also in Gneiting, 2011).

We added a reference to Gneiting (2011) for the relative error. SAPE is not defined in that paper, and we reference the paper by Flores (1986) instead. It seems that the earliest occurrence of the SAPE is due to a book by Armstrong (*Long-range Forecasting: From Crystal Ball to Computer*, 1985); however, as we were unable to access the full text of this book we consider Flores (1986) the more helpful reference.

L138: "$\bar{r}$ is" — Consider $\bar{r}_t$ etc.

L146: "$\bar{\hat{r}}$" — Should be $\hat{\bar{r}}$, or consider $\hat{\bar{r}}_t$.

We applied the suggested changes.

L158 "delta method" — Delta method would seem to also require the distribution to be somewhat tightly distributed, concentrated within a nearly-linear region of the transformation function. But putting significant mass on a wider range of values would also mean having non-negligible mass on negative values if "approximately normal"; it would not make sense to apply the logarithm or sqrt transformations in these cases. So perhaps the "tightly distributed" assumption can be taken. But this seems a bit nonobvious and a justification/application of the delta method would be helpful here.

This argument is a common justification for the choice of variance-stabilizing transformations, see e.g., the below excerpt from *Generalized Linear Models With Examples in R* by Dunn and Smyth:

> Suppose that y has a mean-variance relationship defined by the function $V(\mu)$, with $\text{var}(y) = \phi V(\mu)$. Then, consider a transformation $y^* = h(y) \approx h(\mu) + h'(\mu)(y - \mu)$, from which it can be inferred that
> $$\text{var}(y^*) = \text{var}[h(y)] \approx h'(\mu)^2 \text{var}(y).$$
> Hence, the transformation $y^* = h(y)$ will approximately stabilize the variance if $h'(\mu)$ is proportional to $\text{var}(y)^{-1/2} = V(\mu)^{-1/2}$. When $V(\mu) = \mu^2$ [...], the variance-stabilizing transformation is the logarithm, because then $h'(\mu) = 1/\mu$. When $V(\mu) = \mu$, the variance-stabilizing transformation is the square root because $h'(\mu) = 1/\mu^{1/2}$.

We added a reference to this source. We nonetheless agree with you that this approximation will work best if the distribution is tight and added a short remark to this regard.

The current paragraph reads:

> If the mean-variance relationship of the data-generating distribution is quadratic with $\sigma^2 = c \times \mu^2$, the natural logarithm can serve as the VST (Guerrero, 1993). Denoting by $F_{\log}$ the predictive distribution for $\log(Y)$, we can use the delta method (a first-order Taylor approximation, see e.g., Dunn and Smyth 2018a), to show that

$$\mathbb{E}_F[\text{CRPS}\{F_{\log}, \log(y)\}] \approx \frac{\sigma/\mu}{\sqrt{\pi}} = \frac{\sqrt{c}}{\sqrt{\pi}}. \quad (4)$$

The addition on "tightly distributed" probability mass reads

> We note that while standard in the derivation of variance-stabilizing transformations, the application of the delta method in equations (16) and (17) requires the probability mass of F to be tightly distributed. If this is not the case, the approximation may be less accurate.

L159 — Need to discuss / spell out the point of the above calculation (— that it is the same for all μ, when paired with the same c?).

We added a sentence to make our point clearer:

> As σ and μ are linked through the quadratic mean-variance relationship ($\sigma = \sqrt{c} \times \mu$), the expected CRPS stays constant regardless of the expeccted value of the data-generating distribution μ.

L168: "grows with the variance" — May read like this a linear growth. Consider "grows with the standard deviation".

We have modified the sentence, which now reads:

> We see that when applying the CRPS on the natural scale, the expected CRPS grows monotonically as the variance of the predictive distribution (which is equal to the data-generating distribution for the ideal forecaster) increases.

As the rest of the paragraph talks about the variance, we kept the word variance here as well.

L180: "data-generating" — Consider "fitted"/"believed".

In this instance "data-generating" is correct, as this is about a general property of proper scoring rules. We changed the sentence slightly to make this clearer:

> We computed expected CRPS values for three different distributions, assuming an ideal forecaster with predictive distribution equal to the true underlying (data-generating) distribution.

L186: "incentivizes overconfident predictions" — Needs justifica- tion/citation/removal. This single example disproves propriety but doesn't establish overconfidence as being encouraged in general.

You are right. We removed that sentences as we do not have an analytical result on whether this holds in general.

Figure 2: Were any zeros encountered for the negative binomial or Poisson distributions? Was a shifted log transformation used rather than an unshifted log transformation?

As indicated in the Figure legend ("Expected CRPS was computed on the natural scale (left), after applying a square-root transformation (middle), and after adding one and applying a log-transformation to the data (right)), we have added 1 before applying the log transformation to deal with zero values.

L215: "is mainly driven" — Please add pointer to justification (e.g., Figure 7 / discussion).

We added a reference to Figure 7.

L231: “only include models which” — Consider “only include the 7 models that”

Thank you for the suggestion, we edited the text accordingly.

Figure 5 caption:

- “logarithmic” — With what a values? (THe first link to Figure 5 is from before this information was stated.)
- “D: [. . .] all individual scores” — of EuroCOVIDhub-ensemble or any of the 7 included systems?
- “relative change” — of what change in scores relative to what quantity?

We have updated the caption, which now reads:

> Scores for two-week-ahead forecasts from the EuroCOVIDhub-ensemble (averaged across all forecast dates) for different locations, evaluated on the natural scale as well as after transforming counts by adding one and applying the natural logarithm. D: Corresponding boxplots of all individual scores of the EuroCOVIDhub-ensemble for two-week-ahead predictions. E: Boxplots for the relative change of scores for the EuroCOVIDhub-ensemble across forecast horizons. For any given forecast date and location, forecasts were made for four different forecast horizons, resulting in four scores. All scores were divided by the score for forecast horizon one. To enhance interpretability, the range of visible relative changes in scores (relative to horizon = 1) was restricted to [0.1, 10].

L272: “with the standard deviation” — Also note here that this is for approximately normal data distributions?

We now remind the reader of this assumption by writing

> As (under the simplifying assumption of normality, see Section 2) the WIS/CRPS of an ideal forecaster scales with the standard deviation, a value of $\beta = 1$ would imply a quadratic median-variance relationship;

L282: “independent” — Consider “linearly independent”.

We changed the sentence accordingly.

L288–290: What would stakeholders want this difference to be, and in which direction? I would guess the sqrt would generally be preferable unless applying some extra transformation after the log to re-emphasize locations/times of interest.

That likely depends on the exact purpose stakeholders have in mind. In many instances it is presumably not desirable to give large weight to smaller forecast targets, and one may therefore e.g. choose to use the sqrt transformation or a different one over the log transformation. On the other hand, in our example the log transformation only gives a little bit more weight to smaller forecast targets, which may be acceptable given that the log transformation has nice properties in terms of its interpretation in an epidemiological context.

Figure 7 caption: “one score” — Consider clarifying that/whether there is potentially missingness here.

We have updated the Figure caption to make it more easily understandable. It now reads:

> Correlations of rankings on the natural and logarithmic scale. A: Average Spearman rank correlation of scores for individual forecasts. For every individual target (defined by a combination of forecast

date, target type, horizon, location), one score was obtained per model. Then, for every forecast target, the Spearman rank correlation was computed between scores on the natural scale and on the log scale for all the models that had made a forecast for that specific target. These individual rank correlations were then averaged across locations and time and are displayed stratified by horizon and target types, representing average accordance of model ranks for a single forecast target on the natural and on the log scale. B: Correlation between relative skill scores. For every forecast horizon and target type, a separate relative skill score was computed per model using pairwise comparisons, which is a measure of performance of a model relative to the others for a given horizon and target type that accounts for missing values. The plot shows the correlation between the relative skill scores on the natural vs. on the log scale, representing accordance of overall model performance as judged by scores on the natural and on the log scale.

Figure 8: What is the meaning of the arrow colors? Initially it seems to match the direction, but there is also blue arrow pointing downward. If it is indicating the direction, please fix this and consider a third color for indicating unchanged ranks.

The colour of the error indicates whether or not relative skill scores improved or deteriorated. We agree this was not at all clear and thank you for pointing this out. We updated the plot legend and added a more detailed explanation to the Figure legend:

> Red arrows indicate that the relative skill score deteriorated when moving from the natural to the log scale, green arrows indicate they improved.

The WIS contribution decomposition suggests that an alternative baseline should be used based on differences in transformed data rather than differences in natural scale data. Or, alternatively, that the a value is too low to give reasonable comparisons (as data that is hard/impossible to distinguish from 0 on a plot on the natural scale corresponds with very high penalization of forecasts considering 0 as a possibility). This could (also) be noted in Figure SI.5's caption.

We agree that it would be interesting for the Forecast Hubs to explore baseline models based on transformed differences. However, in this application we aimed for coherence with previous analyses of the same data and thus included the baseline models created within the Forecast Hub in real time.

We also agree it is useful to flag the issues surrounding the selection of a more prominently. We added the following sentence to the caption of Figure SI.5:

> One potential way to do deal with this could be to use a higher a value when applying a transformation $\log(x + a)$, for example $a = 10$ instead of $a = 1$.

We also added a similar sentence to a paragraph in the main text, which now reads:

> A related issue occurs when the predictive distribution has a large probability mass on zero (or on very small values), as this can translate into an excessively wide forecast in relative terms. This can be seen in Figure SI.5. Here, the dispersion component of the WIS is inflated for scores obtained after applying the natural logarithm because forecasts contained zero in its prediction intervals. To deal with this issue one could potentially choose to use a higher a value when applying a transformation $\log(x + a)$, for example $a = 10$ instead of the $a = 1$ that we chose to use.

L340: "convert [...] rate" — Please clarify. Does "multiplicative growth rate" refer to a setup with $\mathbb{E}[X_{t+k}|X_t] = X_t\theta^k$, or only a single step $\mathbb{E}[X_{t+\text{one step}}|X_t] = X_t\theta^k$? Does the proposed conversion apply to both, or only to the latter?

L341–342: "natural logarithm as a [. . .] (VST)" — for what type of relevant distribution(s)?

L344: “take differences between forecasts on the log scale” — Clarify.

L345: “divide each forecast by the forecast” — Caveat about current forecast formats should be included here / in following sentences, without (one mention of) the idea about directly soliciting forecasts of week-on-week ratios in between.

We updated the relevant paragraph which now reads:

> We suggested using the natural logarithm as a variance-stabilising transformation (VST). This is appropriate for variables that are approximately normally distributed and have a quadratic mean-variance relationship with $\sigma^2 = c \times \mu^2$ (this is e.g. approximately true for the negative binomoial distribution and large μ). Alternatively, the square-root transformation can be appropriate in the case of a Poisson distributed variable (Dunn and Smyth, 2018b). Other VST like the Box-Cox (Box and Cox, 1964) are conceivable as well. If one is interested in multiplicative, rather than exponential growth rates, one could, instead of applying a log transformation, convert forecasts into forecasts for the multiplicative growth rate by dividing numbers by the last value that was observed at the time the forecast was made. Forecasters would then implicitly predict a separate multiplicative growth rate from today to horizon 1, 2, etc. Instead of dividing by the last observed value, another promising transformation would be to divide each forecast by the forecast of the previous week (and analogously for observations), in order to obtain forecasts for week-to-week growth rates. Alternatively, one could also take first differences of values on the log scale. This approach would be akin to evaluating the shape of the predicted trajectory against the shape of the observed trajectory (for a different approach to evaluating the shape of a forecast, see Srivastava et al., 2022). Dividing values by the previous value, unfortunately, is not feasible under the current quantile-based format of the Forecast Hubs, as the growth rate of the α-quantile may be different from the α-quantile of the growth-rate. However, it may be an interesting approach if predictive samples are available or if quantiles for weekwise growth rates have been collected. Potentially, the variance stabilising time-series forecasting literature may be a useful source of other transformations for various forecast settings.

L366: “[...] settings” — (to standardize between measurements, then could potentially scale by importance to stakeholders)

We slightly adapted the text to make this point clearer:

> It is possible to go beyond choosing a single transformation by constructing composite scores as a weighted sum of scores based on different transformations. This would make it possible to create custom scores and allow forecast consumers to choose and assign explicit weights to different qualities of the forecasts they might care about.

Figure SI.2: Would benefit from some more discussion, and perhaps a plot of slightly different quantities. E.g., to select an appropriate value of a, it seems like a common goal would be to deprioritize errors around values of x low enough that we expect there to be primarily noise rather than signal or that are not of concern to policymakers. For count data, this likely includes the values 0 - -10 in any circumstances; how far beyond 10 this range extends depends on the range of realistic growth rates, the forecast horizon, and cleanliness of the data. To assist in this decision, it seems like a similar plot of the evaluations of a particular absolute and/or relative error might be useful, or discussion of how to read it from the current plot. E.g., to illustrate that the same relative error is penalized roughly the same for all the plotted values of a for values of x above 50, but quite differently for $a = 10$ vs. $a = 0.1$ for values of x near 1.

We added some more explanation to the caption of Figure SI.2. This makes the trade-off involved in choosing a suitable a value clearer, without prescribing a ’correct’ solution.

> For a given solid line, the dashed line of the same colour marks the x-value that is equal to 5 times the corresponding offset. It can be seen that for a values smaller than one fifth of the transformed

quantity, the effect of adding an offset is generally small. When choosing a suitable a, the trade-off is between staying close to the interpretation of a pure log-transformation (choosing a small a) and not giving excessive weights to small observations (by choosing a larger a, see Figure 3).

Figure SI.7:

- Also specify sorting variable&direction on x axis. (Would a point plot vs. the sorting variable be more informative?)
- Very informative figure, promote to main text? Also would like to see for different “parts” of an epidemic (e.g., steady low periods, increases, peaks/plateaus, decreases). Deaths figures suggest that balancing scores between locations can be tricky, and both cases and deaths suggests that the investigated transformations can’t do this exactly.

We added a statement to the legend ”Locations are sorted according to the mean observed value in that location.” We also followed your suggestion to make this a main figure and added some explanations in the text.

We would expect that one could find a value for a that would eventually yield a more or less flat line, but we haven’t investigated this in practice.

2.2.1 Typographical

L96: “transformtation” → “transformation”

Figure 2 caption: “CRPS values where computed” → “[...] were computed”

L192: “does however influences” → “does however influence”

L249: use two backticks on the left and two single quotes on the right in LaTeX code for the quoting here

Figure 5: “Loaction” → “Location”

Figure SI.7: “Loaction” → “Location”

Thank you very much for pointing these errors out. We have corrected them.

In several places, e.g., L201, there are links to SI material, when clicked, that instead redirect to main text figures.

We’re not sure why this happens and apologise for the confusion. We’re also admittedly not sure how to fix this, but expect this will be dealt with during final typesetting.

3 Reviewer #3

In this manuscript the authors investigate how common scoring rules for probabilistic forecasts can be adjusted by transforming observed data and forecast predictions prior to scoring. They provide three clear motivations for applying such transformations prior to scoring, assess the implications of different transformations in great detail, and evaluate the effects of these transformations using real-world COVID-19

forecasts obtained from the European Forecast Hub. Approaches that can help us improve how we measure the utility of epidemic forecasts for decision-makers are definitely needed. This is a very nice and timely contribution to epidemic forecast evaluation, and I only have a few comments.

Thank you for your kind words.

1. Line 96, typo: "log-transformtation".

We corrected the typo

2. Figure 3: out of curiosity, do the rankings of forecasts A and B first switch at the same observed value (somewhere around 7?) in both panels? Zooming in, it looks as though maybe the switch occurs very slightly earlier for the log-transformed scoring.

You're completely right, on the natural scale, the switch happens between 6.5 and 6.6 and on the log scale between 6.2 and 6.3.

3. Section 3.2, page 9: remarks concerning Figure 4 refer to months (e.g., May, July) but it's a little difficult to relate them to the figure, because the x-axis only has 6-month breaks and spans two years, so it isn't immediately apparent which year (or both?) the reader should focus on. That being said, it's great to see how the log-transformation yields similar scores when the forecasts miss peaks and troughs.

That's a good point, we made the references in the text clearer and added years.

4. Line 281: missing closing parenthesis, "(or $\beta_{\surd} = 0$, respectively".

We corrected the typo

5. Figure 7: I can appreciate that the correlation between the natural and logarithmic scores decreases over the forecast horizon, as the absolute forecast error grows. But I found panel B more challenging to interpret. I gather the key message is that model rankings are quite consistent between the natural and log scales (panel A) even though the absolute scores differ markedly between the two scales — an effect that increases over the forecast horizon (panel B). Perhaps adding a sentence to this effect in the accompanying text (lines 292-295) would help other readers to avoid my confusion.

You are right, we adapted the text and hopefully made it more clear:

> For *individual* forecasts, rankings between models for single forecasts are mostly preserved, with differences increasing across forecast horizons (see Figure 8A). While rankings between forecasters remain similar for a single forecast, this is not true anymore when looking at rankings obtained after averaging scores across multiple forecasts made at different times or in different locations. As discussed earlier, scores on the natural and on the log scale penalise errors very differently, e.g. when looking at performance during peaks or troughs. When evaluating performance *averaged across* different forecasts and forecast targets, relative skill scores of the models therefore change considerably (Figure 8B).

6. Line 296, typo: "Figure Figure 8 shows ...".

Thank you, we corrected the typo.

7. Section 3.4, page 14 and Figure 8: it's a welcome outcome that the Hub ensemble forecast remained the top model, given that the log-transformation clearly affects the individual model rankings. Presumably it would be reasonable to expect that ensemble forecasts for other regions, pathogens, etc, should be the best (or near-best) model when using log-transformed case counts?

While more research is needed to conclude this with more confidence, we generally agree with the statement. We would also expect ensemble forecasts to do well (as they have done in the post) regardless of whether they are evaluated on the log or on the natural scale.

8. Lines 327-329: "A potential downside is that forecast evaluation is unreliable in situations where observed values are zero or very small."

I feel there's an argument to be made here that rather than being a potential limitations, it could be considered an accurate reflection of the inherent uncertainty about the future course of an epidemic when case numbers are very small. This transformation also provides a valuable benefit in these situations, as the authors note a few sentences later: "It also gives higher weight to another type of situation one may care about, namely one in which numbers start to rise from a previously low level". This neatly illustrates the importance (as highlighted by the authors in this manuscript) of using multiple scoring rules to evaluate and compare forecasts.

We think this is a valid point and added a sentence to that effect:

One could argue that this correctly reflect inherent uncertainty about the future course of an epidemic when numbers are small. Users nevertheless need to be aware that this can pose issues in practice.

9. Table SI.2, "Criteria for removing forecasts": forecasts were removed if their median prediction differed greatly from the true value. This should probably be mentioned in the manuscript text, somewhere around line 235 (where Table SI.2 is referenced), to notify the reader that the definition of "erroneous forecasts" includes forecasts that are in extremely poor agreement with the ground truth. Otherwise, I feel that "erroneous" may be open to misinterpretation (e.g., only removing forecasts that predicted negative counts, NaN values, etc).

This is a good point. We changed the sentence to

In addition, we filtered out a small number of erroneous forecasts that were in extremely poor agreement with the observed data, as defined by any of the conditions listed in Table SI.2.

References

Box, G. E. P. and Cox, D. R. (1964). An Analysis of Transformations. *Journal of the Royal Statistical Society. Series B (Methodological)*, 26(2):211–252.

Bracher, J., Ray, E. L., Gneiting, T., and Reich, N. G. (2021). Evaluating epidemic forecasts in an interval format. *PLoS computational biology*, 17(2):e1008618.

Dunn, P. and Smyth, G. (2018a). *Generalized Linear Models With Examples in R.* Springer New York.

Dunn, P. K. and Smyth, G. K. (2018b). *Generalized Linear Models With Examples in R.* Springer.

Dushoff, J. and Park, S. W. (2021). Speed and strength of an epidemic intervention. *Proceedings of the Royal Society B: Biological Sciences*, 288(1947):20201556.

Gneiting, T. and Raftery, A. E. (2007). Strictly Proper Scoring Rules, Prediction, and Estimation. *Journal of the American Statistical Association*, 102(477):359–378.

Good, I. J. (1952). Rational Decisions. *Journal of the Royal Statistical Society. Series B (Methodological)*, 14(1):107–114.

Gostic, K. M., McGough, L., Baskerville, E., Abbott, S., Joshi, K., Tedijanto, C., Kahn, R., Niehus, R., Hay, J., de Salazar, P., Hellewell, J., Meakin, S., Munday, J., Bosse, N. I., Sherrat, K., Thompson, R. N., White, L. F., Huisman, J. S., Scire, J., Bonhoeffer, S., Stadler, T., Wallinga, J., Funk, S., Lipsitch, M., and Cobey, S. (2020). Practical considerations for measuring the effective reproductive number, Rt. *medRxiv*.

Guerrero, V. M. (1993). Time-series analysis supported by power transformations. *Journal of Forecasting*, 12(1):37–48.

Held, L., Meyer, S., and Bracher, J. (2017). Probabilistic forecasting in infectious disease epidemiology: The 13th Armitage lecture. *Statistics in Medicine*, 36(22):3443–3460.

Johansson, M. A., Apfeldorf, K. M., Dobson, S., Devita, J., Buczak, A. L., Baugher, B., Moniz, L. J., Bagley, T., Babin, S. M., Guven, E., Yamana, T. K., Shaman, J., Moschou, T., Lothian, N., Lane, A., Osborne, G., Jiang, G., Brooks, L. C., Farrow, D. C., Hyun, S., Tibshirani, R. J., Rosenfeld, R., Lessler, J., Reich, N. G., Cummings, D. A. T., Lauer, S. A., Moore, S. M., Clapham, H. E., Lowe, R., Bailey, T. C., García-Díez, M., Carvalho, M. S., Rodó, X., Sardar, T., Paul, R., Ray, E. L., Sakrejda, K., Brown, A. C., Meng, X., Osoba, O., Vardavas, R., Manheim, D., Moore, M., Rao, D. M., Porco, T. C., Ackley, S., Liu, F., Worden, L., Convertino, M., Liu, Y., Reddy, A., Ortiz, E., Rivero, J., Brito, H., Juarrero, A., Johnson, L. R., Gramacy, R. B., Cohen, J. M., Mordecai, E. A., Murdock, C. C., Rohr, J. R., Ryan, S. J., Stewart-Ibarra, A. M., Weikel, D. P., Jutla, A., Khan, R., Poultney, M., Colwell, R. R., Rivera-García, B., Barker, C. M., Bell, J. E., Biggerstaff, M., Swerdlow, D., Mier-y-Teran-Romero, L., Forshey, B. M., Trtanj, J., Asher, J., Clay, M., Margolis, H. S., Hebbeler, A. M., George, D., and Jean-Paul Chretien (2019). An open challenge to advance probabilistic forecasting for dengue epidemics. *Proceedings of the National Academy of Sciences*, 116(48):24268–24274.

Lerch, S., Thorarinsdottir, T. L., Ravazzolo, F., and Gneiting, T. (2015). Forecaster's Dilemma: Extreme Events and Forecast Evaluation.

Srivastava, A., Singh, S., and Lee, F. (2022). Shape-based Evaluation of Epidemic Forecasts.

Winkler, R. (1996). Scoring rules and the evaluation of probabilities. *TEST*, 5(1):1–60.
